# Supplementary material for: Direct microsecond wide-field single-molecule tracking and super-resolution mapping via CCD vertical shift
Source: Nat Commun. 2025 Nov 25;16:10503. doi: 10.1038/s41467-025-65529-x (PMC12647137; doi:10.1038/s41467-025-65529-x)
Supplement: Supplementary file 3 — Description of Additional Supplementary Files [file 41467_2025_65529_MOESM3_ESM.pdf]

Description of Additional Supplementary Files

Supplementary Video 1. Example SpeedyTrack data frames of Cy3B-labeled carbonic anhydrase diffusing in PBS, with an exposure time of 300 µs and vertical shift time of 7.5 µs for 15 rows at each timepoint. Left, center, and right panels show SpeedyTrack frames recorded at the beginning, middle, and end of one run, with frame numbers marked at the top. Scale bar: 5 µm.

Supplementary Video 2. Example VS-SpeedyTrack data frames of Dendra2 FP diffusing in the ER lumen of a living COS-7 cell, performed at 500 µs resolution. Left, center, and right panels show VS-SpeedyTrack frames recorded at the beginning, middle, and end of one run, with frame numbers marked at the top. Scale bar: 5 µm.
